# Multidisciplinary Management of Pediatric Hepatoblastoma: A 20-Year Single-Center Experience

**DOI:** 10.5152/tjg.2022.21827

**Published:** 2022-12-01

**Authors:** Funda Tayfun Küpesiz, Ayşe Nur Akınel, Hilal Akbaş, Çiğdem Sivrice, Gülen Tüysüz Kintrup, Güngör Karagüzel, Mustafa Melikoğlu, Mustafa Tekinalp Gelen, Bülent Aydınlı, Alphan Küpesiz, Elif Güler

**Affiliations:** 1 Division of Pediatric Hematology, Oncology and BMT, Department of Pediatrics, Akdeniz University Faculty of Medicine, Antalya, Turkey; 2 Department of Hematology-Oncology, Clinic of Pediatrics, Konya City Hospital, Konya, Turkey; 3 Department of Pediatric Surgery, Akdeniz University Faculty of Medicine, Antalya, Turkey; 4 Department of Pathology, Akdeniz University Faculty of Medicine, Antalya, Turkey; 5 Division of Organ Transplantation, Department of General Surgery, Akdeniz University Faculty of Medicine, Antalya, Turkey

**Keywords:** Chemotherapy, hepatoblastoma, liver transplantation, PRETEXT, radiofrequency ablation, surgical resection

## Abstract

**Background:**

Hepatoblastoma is rare cancer that responds well to risk-based chemotherapy, and surgical treatment is needed to achieve complete remission and satisfactory survival rates in hepatoblastoma patients. In this study, we evaluated the clinical features and treatment outcomes of pediatric hepatoblastoma patients treated in our clinic.

**Methods:**

Eighteen patients with hepatoblastoma who were treated and followed up in our center between June 1999 and June 2020 were analyzed retrospectively. All patients were evaluated by a multidisciplinary team and managed using a risk-based protocol (SIOPEL-1 and SIOPEL-3).

**Results:**

The patients’ mean age at diagnosis was 38.33 ± 52.34 months. Sixteen patients (89%) received neoadjuvant chemotherapy, and 2 patients (11%) who underwent complete mass excision at diagnosis received adjuvant chemotherapy. After neoadjuvant therapy, the tumor was completely resected in 8 patients (45%), while liver transplantation was performed in 6 patients (34%) because complete resection of the tumor was not possible. Two patients died before surgical treatment. One patient relapsed with lung metastasis after salvage chemotherapy. She is alive without disease at 64 months. The mean follow-up time was 59.3 ± 49.8 months; 5-year overall and disease-free survival rates were 88.9% and 80.8%, respectively. The 5-year overall survival rate was 100% for both liver transplant and resected patients, whereas 5-year disease-free survival was lower in transplant patients (75% vs 100%, *P *< .001).

**Conclusion:**

Multidisciplinary follow-up is especially important for patients who may need liver transplantation. Some patients may benefit from new treatment options such as radiofrequency ablation and cyberknife treatment.

**Main Points**

- Liver transplantation has an important role in the surgical management of pediatric hepatoblastoma (HB) patients presenting with multifocal or metastatic disease.
- Our data indicate that liver transplantation is an effective treatment for pediatric patients with HB.
- Among pediatric HB patients who received chemotherapy, there was no difference in overall survival between those who underwent liver transplant and surgical resection, while disease-free survival was better after surgical resection.
- In the treatment of pediatric patients with multifocal or metastatic HB, early multidisciplinary evaluation contributes to surgical planning and selection of alternative treatment methods, thereby improving long-term survival.

## Introduction

Hepatoblastoma (HB) is the most common primary malignant liver tumor in children (50%-60% of all hepatic tumors), although it accounts for only 1% of all pediatric cancers.^1^ The annual incidence of HB is 1.5 per million children, and it occurs more frequently in children younger than 3 years of age.^2^ Initial presenting complaints are typically mass-related abdominal pain, swelling and distension, loss of appetite, and malaise. Rarely, patients may present with acute abdomen, peritonitis, and severe anemia due to tumor rupture.^1,3^

Ultrasonography is an easy, accessible, and inexpensive imaging method. However, computed tomography (CT) and magnetic resonance imaging (MRI) help determine tumor location, segmental extension, and proximity to hepatic vessels, with spiral CT being particularly helpful in the imaging of hypervascular lesions.^4^ The pre-treatment extent of disease (PRETEXT) staging system, defined by the International Childhood Liver Tumours Strategy Group (SIOPEL), evaluates the pretreatment extent of disease based on preoperative radiological imaging and is of strategic importance in treatment planning.^5^

The only viable treatment for HB that offers long-term disease-free survival (DFS) is complete surgical resection. However, fewer than 50% of HB patients have tumors amenable to complete resection at the time of diagnosis.^6^ With neoadjuvant chemotherapy, the tumor shrinks and becomes hard and fibrotic, resulting in 87% of patients being eligible for surgery.^7,8^

Prognosis is poor for patients with recurrence in the residual liver after resection.^9,10^ Therefore, if the tumor would not be completely removed, liver transplantation should be considered as the primary option when selecting surgical treatment.^10^

Although international or organization-based protocols greatly contribute to the patient management, the data related to national or center-based modifications of these protocols are also important. This study aimed to evaluate the clinical features of HB patients treated in our center over the last 20 years and the results of our treatment protocol in the light of the literature.

## MATERIAL AND METHODS

Patients with malignant liver tumors who were treated and followed up in our center between June 1999 and June 2020 were evaluated retrospectively. Patients diagnosed as having hepatocellular carcinoma (n = 5) and rhabdoid tumor (n = 1) were excluded from the study.

The patients were evaluated according to PRETEXT staging using dynamic CT or CT imaging.^11^ In addition to PRETEXT stage, extrahepatic extension of the tumor was noted in terms of caudate lobe involvement (C), extrahepatic abdominal tumor (E), presence of multifocal tumor (F), tumor rupture or intraperitoneal bleeding (R), presence of distant metastasis (M), lymph node metastases (N), portal vein involvement (P), and vena cava or hepatic vein involvement (V).

Follow-up and treatment were performed in accordance with the SIOPEL-1 and SIOPEL-3 protocols. Patients with PRETEXT -III tumors without metastasis or extrahepatic findings (V, P, E, R, and F) were classified in the standard-risk group. Patients with PRETEXT IV and/or lung metastasis, intra-abdominal spread, tumor rupture at admission, or alpha-fetoprotein (AFP) level below 100 ng/mL were included in the high-risk group. Serum AFP levels were measured at the time of diagnosis and before each course of chemotherapy. The patients were evaluated for surgical treatment after 2 courses of chemotherapy. Those whose tumors were not amenable to complete resection were reevaluated after another 2 courses of chemotherapy. Decisions regarding tumor resection or liver transplantation were made by a multidisciplinary team. Orthotopic liver transplantation was performed for patients with unresectable tumors and no metastasis.

The patients’ demographic data (age and sex), histopathological diagnosis, AFP level at the time of diagnosis, PRETEXT stage, metastasis status, preoperative chemotherapy, surgical procedure, and recurrence information were obtained from the patient charts and hospital data archives. The study was approved by the Akdeniz University Faculty of Medicine Clinical Research Ethics Committee (70904504/22).

### Statistical Analysis

The data were statistically analyzed using IBM Statistical Package for the Social Sciences version 23.0 software package (IBM Corp, Armonk, NY, USA). Descriptive statistics were presented as mean ± standard deviation and median (minimum-maximum) values. Intergroup quantitative variables were compared using Student’s *t*-test for parametric data and the Kruskal–Wallis and Mann–Whitney *U* tests for nonparametric data. Qualitative variables were analyzed using Kruskal–Wallis, Mann–Whitney *U* test, chi-square, and Fisher’s exact tests as appropriate. Results with a *P* value less than .05 were considered statistically significant.

## Results

A total of 18 children (female to male ratio = 0 : 63) diagnosed as having HB in our pediatric oncology department between 1999 and 2020 were included in the study. The mean age at diagnosis was 38.33 ± 52.34 months (median= 14 month, range = 6 days-170 months). None of the patients had risk factors associated with HB (in vitro fertilization, prematurity, very low birth weight, Beckwith-Wiedemann syndrome).

Abdominal pain and abdominal swelling were the most common presenting complaints (n = 7). In 3 patients, the liver mass was detected incidentally. Other presenting complaints were elevated liver enzymes and jaundice (n = 2), fever (n = 2), and diarrhea (n = 2). In the other 2 patients, the mass was detected antenatally and they were referred to the pediatric oncology department after birth. The median AFP level at diagnosis was 1.12 × 10^5^ ng/mL (range = 30-1.12 x 10^6^). Two patients presented with AFP lower than 100 ng/mL.

Tissue diagnosis was established by histopathological examination in all patients. At diagnosis, 2 patients (11%) underwent complete surgical resection and the remaining 16 patients (89%) underwent biopsy. The subtype of HB could not be determined for 11 patients (61%), while 3 patients (17%) had an epithelial type and 3 (17%) had mixed mesenchymal-epithelial HB. One patient (5.5%) was diagnosed with small cell undifferentiated HB. Three patients had Stage I (17%), six patients had Stage II (33%), four patients had Stage III (22%), five patients had Stage IV (28%). Metastases were located in the lung in all 3 patients (17%) with distant organ metastasis.

The characteristics of our cohort grouped by standard-risk and high-risk HB are detailed in Table 1. Of the patients, 44% (n = 8) were in the standard-risk group and 56% (n = 10) were in the high-risk group.

## Treatment Characteristics

**Figure 1.**
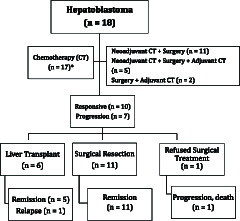
Flowchart of the treatments and outcomes of patients with hepatoblastoma. ^*^One patient died without receiving chemotherapy or surgery due to tumor rupture at the start of the first course of chemotherapy.

Four patients (22%) were treated according to the SIOPEL-1 protocol and 14 (78%) according to the SIOPEL-3 protocol. Four patients treated according to the SIOPEL-1 protocol received PLADO (cisplatin and doxorubicin) therapy independent of their risk group. Since total resection of the tumor was performed in the surgical procedure performed for diagnostic sampling in 2 of these patients, the protocol was modified and neoadjuvant treatment was not applied to the patients. Six courses of adjuvant therapy were given to these patients (Table 1, Patients 1 and 3). In the SIOPEL-3 protocol, patients in the standard-risk group (n = 6) received cisplatin monotherapy and patients in the high-risk group (n = 8) received SuperPLADO (cisplatin, doxorubicin, and carboplatin). Sixteen patients (89%) were started on neoadjuvant chemotherapy according to their protocol: PLADO (n = 3), cisplatin monotherapy (n = 6), and SuperPLADO (n = 7) (Table 1). The treatment flowchart is shown in Figure 1.

The AFP value regressed with chemotherapy in all patients except patient 13. AFP basal values of patients and AFP levels at the time of treatment response evaluation are shown in Table 1.

Of the patients in the standard-risk group who received cisplatin monotherapy, 3 patients did not show sufficient tumor reduction for complete surgical resection and continued treatment with SuperPLADO. Two of those patients later underwent complete tumor resection and 1 underwent liver transplantation. In the high-risk group, all patients who started SuperPLADO as neoadjuvant chemotherapy showed an adequate response after 2 courses.

One of the patients who initially received PLADO according to the SIOPEL-1 protocol was switched to ICE (ifosfamide, carboplatin, and etoposide) rescue therapy due to inadequate response at the interim evaluation and was followed up disease-free after hepatectomy (patient 10).

The patient who was diagnosed neonatally and had high-risk HB (patient 18) received SuperPLADO chemotherapy and the mass was reduced enough for surgical treatment. However, the family refused surgery or further chemotherapy and terminated treatment. The patient died 5 months after diagnosis due to disease progression.

Surgical resection was performed in 2 patients at diagnosis (patients 1 and 3) and in 15 patients when it was deemed appropriate. The operations performed were right or left hepatectomy (n = 5), segmentectomy (n = 6), and orthotopic liver transplantation (n = 6). One of the transplant patients was in the standard-risk group and 5 were in the high-risk group. Liver transplantation was performed from living donors in 5 cases and deceased donors in 2 one cases. In all patients with lung metastases (patients 11, 13, and 16), pretransplant evaluation demonstrated that the lung metastases had disappeared.

In 1 patient, liver transplantation was planned because mass excision was not possible at the time of primary surgery, but a suitable living donor could not be found (patient 14). This unresectable patient also had elevated AFP, so their chemotherapy protocol was switched to C5V (cisplatin, 5-fluorouracil, and vincristine). A decrease in AFP level was observed during follow-up, and the patient underwent transplantation from a deceased donor.

In 1 patient with an unresectable tumor due to tumor location, surgery was completed after performing intraoperative radiofrequency ablation (RFA).

One patient (patient 13) with AFP level below 100 ng/mL at admission relapsed with lung metastasis 34 months after diagnosis. Following salvage therapy, the patient remains in remission at 63 months. Two patients underwent treatment by CyberKnife due to lung metastases.

### Survival Analysis

The mean follow-up time of the patients in the study was 59.3 ± 49.8 months (median = 52 months, range = 3 days to 168 months). Except for 2 high-risk patients who died (11%), all patients (89%) are in remission and under follow-up. One of the nonsurviving patients died due to intratumoral hemorrhage at the beginning of the first course of chemotherapy. The other patient was diagnosed in the neonatal period and the family refused surgery.

Relapse was observed in only 1 patient (5.5%) in our cohort (patient 13). After undergoing liver transplantation, the patient’s AFP level increased again and lung metastasis was detected. The patient underwent rescue chemotherapy and CyberKnife treatment for lung metastasis and is currently under follow-up and disease-free at 63 months.

**Figure 2.**
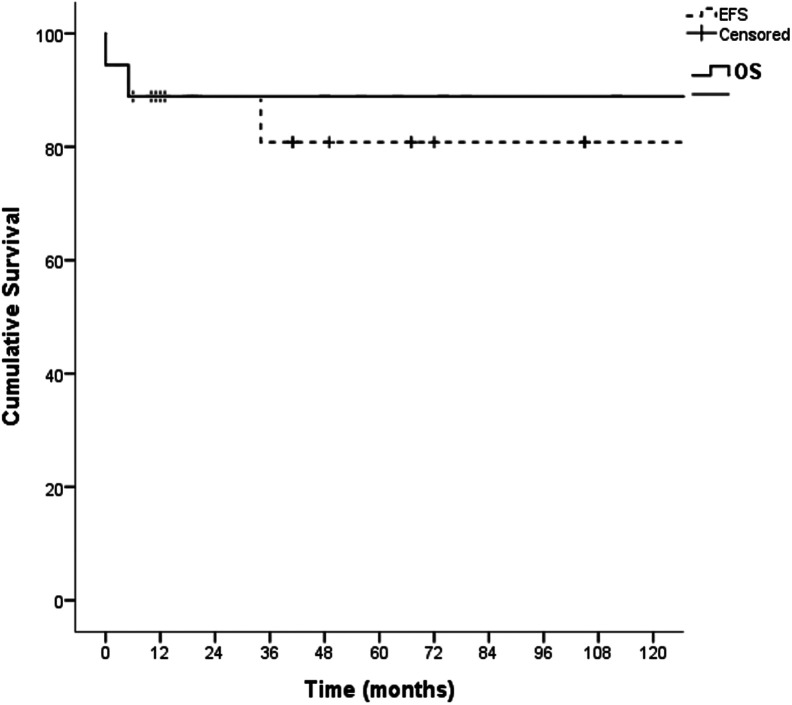
The 5-year overall rates and event-free rates.

The 5-year overall (OS) and DFS rates were 88.9% and 80.8%, respectively (Figure 2).

Five-year DFS was 92.9% for patients aged ≤5 years, 100% for patients aged >5-10 years, and 50% for patients aged >10 years, although the difference was not statistically significant (*P *= .103). There was no difference in OS between the age groups.

**Figure 3.**
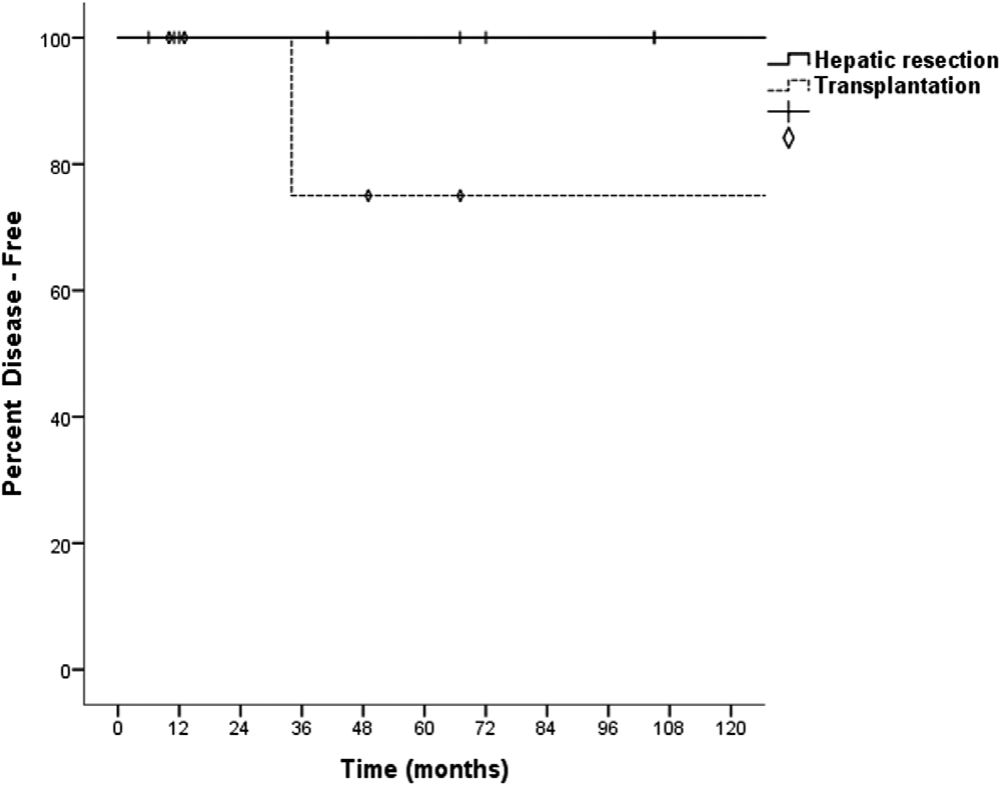
The 5-year disease-free survival curves of patients with liver transplantation versus hepatic resection.

In terms of surgical treatment, the 5-year OS rate was 100% for patients who underwent liver transplantation and those who underwent primary surgical resection (*P *> .05). The 5-year DFS rate was 75% for liver transplant patients and 100% for primary surgical resection patients (*P *< .05) (Figure 3).

Sex, risk group, disease stage, presence of metastasis, and AFP level were not associated with OS or DFS (*P *> .05).

## Discussion

Before the 1980s, HB was treated surgically. After discovering that HB is chemosensitive, the increase in survival with chemotherapy regimens containing cisplatin and doxorubicin marked a turning point in treatment.^12,13^

Different pediatric oncology groups vary in terms of their diagnostic and treatment approaches to patients with HB. Some groups have stated that in patients 6 months to 3 years of age with elevated AFP, treatment can be initiated without biopsy due to the risk of tumor rupture and dissemination.^14^ However, the Japanese Study Group for Pediatric Liver Tumor (JPLT) insists on a histopathological diagnosis unless there is a life-threatening condition such as tumor rupture or tumor invasion into the right atrium.^15^ A 5-year-old patient who was referred to our department with radiologically suspected HB and high AFP value was diagnosed by biopsy as having liver hamartoma, while a 17-month-old patient with AFP level of <100 ng/mL was diagnosed as having rhabdoid tumor by demonstrating INI1 loss. Due to this experience, HB diagnosis was confirmed histopathologically in all patients in this series before initiating chemotherapy. Hepatoblastoma usually occurs in the first 3 years of life, as in this series.^16^ The median age at diagnosis was 14 months and 60% of the patients were male (11/18), which is consistent with the literature. 

The Children’s Oncology Group (COG) and JPLT recommend primary surgical resection followed by adjuvant chemotherapy based on staging and risk factor assessment for patients with PRETEXT I-II disease.^17,18^ SIOPEL and German Society of Pediatric Oncology and Hematology (GPOH) recommend a treatment plan of preoperative (neoadjuvant) chemotherapy, primary surgical resection, and postoperative (adjuvant) chemotherapy due to the higher surgery-related mortality in patients who do not receive neoadjuvant chemotherapy.^5,18,19^

The Children’s Hepatic Tumors International Collaboration workshop evaluated the approaches of SIOPEL, COG, GPOH, and JPLT and their treatment results between 1985 and 2008. Apart from PRETEXT stage, risk stratification was determined considering M, V, P, E, R, and F involvement and reduction in AFP.^2,18-20^

Patients in the present series were treated according to SIOPEL protocols. Despite significant progress in the treatment of children with localized HB, the prognosis for patients with metastatic disease in the SIOPEL-1 study was 28% DFS and 57% OS at 5 years.^21^ In our series, we are following 4 patients who were treated according to the SIOPEL-1 protocol are currently disease-free. Of the 14 patients whose treatment was planned according to the SIOPEL-3 protocol, 12 survived and are currently disease-free (5-year OS rate of 85.7%). The 2 patients who died during SIOPEL-3 treatment (1 due to tumor rupture and hemorrhage and the other due to treatment refusal) cannot be considered failures of this protocol. Retrospective evaluation of the patients in our series showed that 4 patients met the criteria for the very high-risk group in the SIOPEL-4 protocol. Of these, 2 patients showed good response to treatment with SuperPLADO and are still in remission after liver resection, 1 underwent liver transplantation after their lung metastases disappeared, and the other patient died due to tumor rupture during the first course of chemotherapy. Although the patients did not receive the intensified chemotherapy regimen as in SIOPEL 4, treatment with SuperPLADO controlled the disease enough for them to be eligible for surgical treatment.

Surgery is the cornerstone of HB treatment, and complete surgical resection is essential for survival. However, resection is not possible at the time of diagnosis in 70%-80% of cases because the tumor is too large, multifocal, or too close to major vascular structures. With neoadjuvant chemotherapy, the tumor resection rate in these cases increases to 80%. Nevertheless, 20% of these patients require liver transplantation because curative surgical resection is not possible.^20-22^ In our series, 2 patients (11%) underwent surgical resection at the time of diagnosis. After neoadjuvant chemotherapy, tumors were resectable in 68.7% of the patients.

In the literature, liver transplantation is recommended for nonmetastatic disease that is not amenable to surgical resection.^18,23^ Otte et al^24^ reported that the 6-year DFS rate was better in those who underwent primary liver transplantation (82%) than in those who underwent rescue liver transplantation (30%). In our series, patients for whom surgical resection was not possible despite chemotherapy (33.3%) underwent liver transplantation. This rate is higher than in the literature, which may be related to the fact that 50% of the patients had PRETEXT III and IV disease and liver transplantation can be performed in our center.

Shanmugam et al^25^ used PLADO and SuperPLADO regimens as chemotherapy in their series of 30 HB patients and performed liver transplantation in 20% of those patients. The patients who did not respond to chemotherapy (17%) died without undergoing surgical treatment. They reported a DFS rate of 60% in the high-risk group and 93% in the standard-risk group during the 30-month follow-up.^25^ Tezer Kutluk et al^26^ detected distant organ metastasis in 16% of 91 children with HB children in their liver tumor study. Similarly, the proportion of patients with distant organ metastasis in our study was 16.6%. PRETEXT I/II patients accounted for 34% of their patient population and 50% of our patients. Although their study was also conducted in Turkey, they reported a 5-year OS rate of 32.4% for all patients, while the 5-year OS rate in our study was 88.9%. In our series, the 5-year DFS rate was 64% in the high-risk group and 100% in the standard-risk group. None of our patients underwent surgery due to nonresponse to chemotherapy. Only the 2 nonsurviving patients could not undergo surgical treatment.

In a study evaluating 87 children with HB treated in a single center in China, the 1- and 5-year mean survival rates were 87.7% and 78.9%, respectively.^27^ In this study, male gender was found to be an independent risk factor associated with poor prognosis, while surgical treatment (hepatectomy or liver transplantation) was independently associated with good prognosis. The negative impact of male sex on prognosis was also reported in another study.^28^ In our series, there was no difference in DFS or OS rates based on patient sex. However, due to the small number of patients, we are not able to make a clear inference on this subject.

Although there was no statistically significant difference among the age groups, the 5-year DFS rates were 92.9% for patients aged ≤5 years, 100% for patients aged >5-10 years, and 50% for patients aged >10 years (*P *= .102). The lack of statistical significance may have been due to the small number of patients in our series. A National Cancer Institute analysis of a cohort from 2004 to 2016 evaluated 443 children with HB and showed that the risk of death was lowest in patients under 1 year of age and highest in those aged 5-18 years.^10^

Serum AFP level is the most important clinical marker used in diagnosis, treatment response, and detection of recurrence. It should be kept in mind that patients with very low AFP levels (<100 ng/mL) may have small cell undifferentiated type or rhabdoid tumors. Both of these histological types have a poor prognosis.^29^ In pediatric hepatic tumors, histological type was found to be associated with prognosis regardless of staging. COG reported a 5-year DFS rate of 100% for patients with fully resected PRETEXT-I tumors with pure fetal histology.^17^ In this series, the 1 patient who had AFP <100 ng/mL was diagnosed as having small cell undifferentiated HB. The only relapse in our series was in this patient, who developed pulmonary relapse after liver transplantation. The patient is currently disease-free 63 months after rescue chemotherapy and CyberKnife treatment for lung metastases.

In cases where complete removal of the mass is not possible after neoadjuvant chemotherapy, reducing the mass with hepatic artery chemoembolization (TACE) may allow surgical resection to be performed.^30^ In 2008, Ye et al^31^ reported a 2-year-old patient who had recurrence after liver resection and was cured by percutaneous RFA. Although the number of cases is small, RFA appears to be a promising treatment option in children with recurrent HB.^32-34^ However, its role in the treatment of primary HB remains unknown. In our series, 1 patient for whom surgical resection was still considered risky after neoadjuvant chemotherapy became eligible for surgical resection after intraoperative RFA and is still disease-free at 79 months.

This study had several limitations. First, this was a retrospective, single-center study. Nevertheless, the quality of the collected data is high because all data were reviewed and updated. Second, the number of HB patients was small, but this series is valuable as HB is a rare tumor of childhood. Although the number of cases is statistically limited to be able to make subgroup analyses, the cases evaluated provide an idea as they reflect the multidisciplinary treatment approach.

## Conclusion

Our patients’ outcomes are comparable to those reported in the literature. It should be kept in mind that a multidisciplinary approach is important in these patients and that liver transplantation will increase survival, especially in cases where tumor location precludes complete resection, and organ transplant surgeons should evaluate these patients in this regard in the early period.

## Figures and Tables

**Table 1.** Patients’ Clinical Characteristics, Disease Status, Chemotherapy, Surgery, and Treatment Outcomes

Patient Number	**Age (Months)**	**Sex**	**PRE TEXT**	**Protocol**	**Alpha-Fetoprotein Level (ng/mL)**		**Chemotherapy**		**Primary Surgical Treatment**	**Final Status**	**Follow-Up Time (Months)**
					**Baseline **	**After Neoadjuvant Treatment**	**Neoadjuvant** **(Number of Courses)**	**Adjuvant** **(Number of Courses)**			
Standard-Risk Hepatoblastoma											
1.	2	M	2	SIOPEL 1**^¥^**	2993	21^**^	No	PLADO (6)	Right trisegmentectomy	Alive without disease	112
2.	83	M	2	SIOPEL 1	16 470	50	PLADO (4)	PLADO (2)	Right lobectomy + CyberKnife	Alive without disease	112
3.	10	M	1	SIOPEL 3**^¥^**	128 121	835^**^	No	Cisplatin (6)	Left lateral segmentectomy	Alive without disease	13
4.	11	M	2	SIOPEL 3	60 500	90	Cisplatin (6)	No	Left lobectomy	Alive without disease	74
5.	52	F	1	SIOPEL 3	448	4.9	Cisplatin (4)	Cisplatin (2)	Right monosegmentectomy	Alive without disease	48
6.	11	M	3	SIOPEL 3	181 920	13.4	Cisplatin (4) + SuperPLADO (2)	No	Living-donor liver transplantation	Alive without disease	20
7.†	6 days	M	1	SIOPEL 3	1 118 070	13.8	Cisplatin (5) + SuperPLADO (1)	SuperPLADO (3)	Left lateral segmentectomy	Alive without disease	48
8.	7	M	3	SIOPEL 3	144	31.3	Cisplatin (2) + SuperPLADO (7)	No	Right lobectomy + RFA	Alive without disease	79
High-Risk Hepatoblastoma											
9.	27	F	2V	SIOPEL 1	96 800	1412	PLADO (6)	No	Living donor liver transplantation	Alive without disease	148
10.	7	M	4	SIOPEL 1	121 000	79000	PLADO (4) + ICE (2)	ICE (4)	Extended left hepatectomy	Alive without disease	168
11.	14	F	2M	SIOPEL 3	161 000	58	SuperPLADO (6) + C5V (8)	No	Deceased donor liver transplantation	Alive without disease	74
12.	73	F	4N	SIOPEL 3	14 215	1894	SuperPLADO (7)	No	Living donor liver transplantation	Alive without disease	56
13.*	170	F	4M	SIOPEL 3	30	5843	SuperPLADO (6)	Superplado (10) + C5V (8) + CyberKnife + ICE (3)	Living donor liver transplantation	Alive without disease after relapse	64
14.	28	F	2CV	SIOPEL 3	199 345	0,91	SuperPLADO (5)	No	Posterior segmentectomy	Alive without disease	18
15.	17	M	3N	SIOPEL 3	192 032	11	SuperPLADO (4)	SuperPLADO (1)	Left hepatectomy	Alive without disease	19
16.*	14	M	4M	SIOPEL 3	30	29	SuperPLADO (5)	No	Living donor liver transplantation	Alive without disease	17
17.	163	M	3N	SIOPEL 3	787 000	Not approved	SuperPLADO (1)	No		Dead with disease	3 days

^*^AFP at diagnosis <100 ng/mL.

^†^Diagnosed neonatally.

^¥^The SIOPEL 1 protocol was modified in patients 1 and 3 who underwent complete mass excision at the time of diagnosis and neoadjuvant therapy was not applied. These patients received only adjuvant therapy.

^**^AFP values after adjuvant chemotherapy.

PRETEXT, PRETreatment EXTent of disease; PLADO, cisplatin, doxorubicin; SuperPLADO, cisplatin, doxorubicin, carboplatin; ICE, ifosfamide, carboplatin, etoposide; C5V, cisplatin, 5-fluorouracil, vincristine; RFA, radiofrequency ablation; RT, related donor transplant; URT, unrelated donor transplant.
